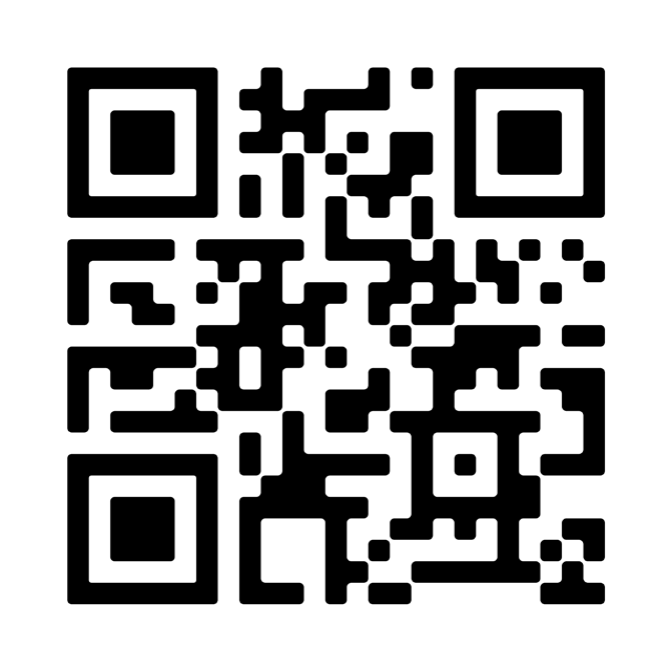# Laparoscopic cornual resection for interstitial pregnancy: Staying in the Marginal Zone

**DOI:** 10.52054/FVVO.16.3.032

**Published:** 2024-09-30

**Authors:** N Kathopoulis, K Kypriotis, A Douligeris, D Zacharakis, A Prodromidou, I Chatzipapas, T Grigoriadis, A Protopapas

**Affiliations:** 1st Department of Obstetrics and Gynecology, University of Athens, Alexandra hospital, Athens, Greece

**Keywords:** Cornual resection, ectopic pregnancy, laparoscopy

## Abstract

**Background:**

Fortunately, interstitial pregnancies are a rare early pregnancy presentation, yet they can be challenging to managed and are associated with a high risk of intra-abdominal haemorrhage. Once detected, surgical laparoscopic resection can be the preferred management method for both patient safety and for definitive treatment.

**Objective:**

The video presents a new technique for laparoscopic resection of an interstitial pregnancy which enables the procedure to be effectively bloodless.

**Materials and Methods:**

We report on a new technique for laparoscopic cornual resection. As shown in the video, staying in the marginal zone may result in the enblock resection of the gestational sac. Using meticulous applications of bipolar energy and cutting with scissors in the marginal zone, the operation may be completed with almost no blood loss and minimal damage to the adjacent healthy myometrium.

**Results:**

The operation lasted 30 min with almost no blood loss. The patient had an uneventful recovery and was discharged on the first postoperative day.

**Conclusions:**

Staying in the marginal zone during dissection permits even less experienced laparoscopists to complete laparoscopic cornual resection with minimal blood loss concomitantly with minimal trauma to the adjacent myometrium.

## Learning objective

To minimise intra-operative blood loss, surgeons should aim to stay in the marginal zone when performing laparoscopic cornual resection for interstitial pregnancy.

## Introduction

Whilst interstitial pregnancies only accounts for 2-4% of all ectopic pregnancies, this rare pathology can be challenging to diagnose and can be associated with massive haemorrhage and adverse sequela to the patient (Bhagavath and Lindheim, 2021). Early diagnosis is imperative for proper management, and high-end transvaginal ultrasonography combined with adequate expertise has improved this early diagnosis.

The majority of these cases are treated either conservatively with expectant management or medically, mainly with methotrexate injection (Jermy et al., 2004). Nevertheless, this drug may result in serious side effects such as hepatorenal dysfunction and patients may go on to require secondary surgical treatment (Moawad et al., 2010). For some patients, surgical management can be the primary treatment. With advancements in equipment and in surgical technique, laparoscopy offers a safe approach to the management of interstitial pregnancies (Marchand et al., 2021). We report a new technique of laparoscopic cornual resection that is simple, reproducible, and highly effective for minimising intraoperative blood loss.

## Patients and methods 

**Figure 1:**
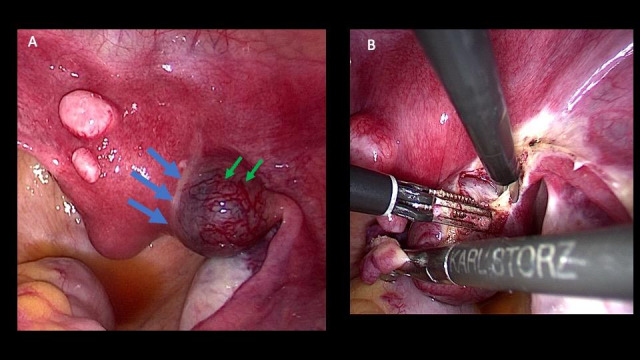
(A) Interstitial pregnancy with the marginal zone between thick myometrium and thin myometrial layer covering the gestational sac (blue arrows). Notice the markedly dilated vessels (green arrows). (B) Cornual resection starts from the mesosalpingeal part after resecting the tube from its fimbrial end.

**Figure 2:**
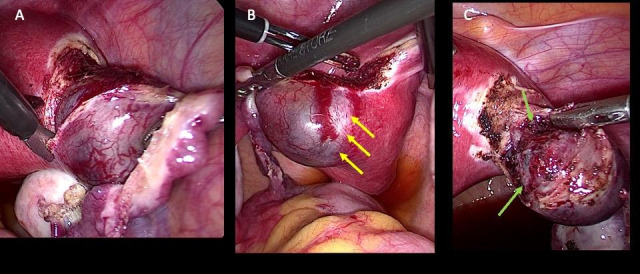
(A) Staying in the marginal zone, there is minimal blood loss, and the gestational sac is enucleated intact from the myometrium. (B) Marginal zone on the posterior uterine wall (yellow arrows). (C) Intact gestational sac almost removed from the uterus (green arrows).

A 35-year-old patient with a history of two caesarean sections presented to our unit with a seven-week ectopic pregnancy. The transvaginal ultrasound revealed a 3cm gestational sac within the right cornua and the serum βhCG was 15,342 IU/mL. The patient was scheduled for laparoscopic cornual resection. Under general anaesthesia, the patient was placed in a lithotomy position and 12mmHg pneumoperitoneum was established using the Veress needle. After the insertion of the 10mm umbilical trocar, the scope was introduced and two 5mm lateral and one suprapubic trocar were placed under vision. Intraoperatively, a right interstitial pregnancy with markedly dilated vessels over the surrounding myometrium was revealed (Figure 1A). Initially, it is our standard practice to recognise the marginal zone among the dense myometrium and the thin myometrial layer covering the gestational sac, which will be the future cleavage plane. Following this technique, no vasoconstricting agent is needed to reduce blood loss. The operation starts with mesosalpinx dissection from the fimbria up to the interstitial segment of the tube (Figure 1B). Staying within the marginal zone by dividing it circumferentially with intermittent applications of bipolar energy and scissors will result in intact gestational sac enucleation and minimal damage to the surrounding myometrium. Staying in the marginal zone is imperative as if the cleavage plane is proximal to it, a higher portion of healthy myometrium is resected resulting in more bleeding (Figure 2A-C). Moreover, cutting distally to the marginal zone results in damaging the capsule of the gestational sac predisposing to possible retention of products and bleeding. The defect is sutured in one or two layers depending on its depth to ensure restoration of uterine anatomy and reduce the potential for uterine rupture in any subsequent pregnancies. The gestational sac and the right tube were removed enblock and sent to the pathology department for analysis.

## Results

The operation lasted 30 min with almost no blood loss. The patient had an uneventful recovery and was discharged on the first postoperative day. Follow-up β-hCG became undetectable 13 days after the surgery. No adjunct therapy was required.

## Discussion

The surgical management of interstitial pregnancies continues to be a therapeutic dilemma. Most cases are treated with expectant or medical management, mainly with methotrexate injection. When surgical treatment is decided, cornual resection by laparotomy was the established traditional approach, but recently, minimally invasive surgery has been applied for this rare pathology. Various techniques have been described to ensure the safe completion of laparoscopic cornual resection to reduce intraoperative blood loss, which is the surgeon’s primary concern. Many authors have proposed administering haemostatic pharmaceutical agents such as vasopressin, misoprostol, and tranexamic acid (Kathopoulis et al., 2022; Protopapas et al., 2019; Protopapas et al., 2020).

Moreover, different prophylactic sutures, such as purse-string, encircling, and endo-loop sutures, have been applied to act as a tourniquet and reduce blood loss (Afifi et al., 2016; Min et al., 2023). Temporary uterine artery ligation is another surgical manoeuvre commonly used for the same purpose (Zheng et al., 2021). These haemostatic techniques may be used individually or in combination, sometimes increasing the complexity of the surgery and possible complications. Some of the techniques also demand sophisticated instrumentation and an experienced laparoscopic surgeon.

Our technique described in the video is simple, easily reproducible, and safe. Using simple instrumentation (bipolar energy and scissors) with no advanced surgical skills required the surgeon can perform this demanding surgery. Staying in the marginal zone during dissection is crucial in achieving haemostasis and minimising the risk of damaging the healthy myometrium. Following our technique, the gestational sac can be extracted intact, resulting in minimal intraoperative blood loss which is the main goal of laparoscopic cornual resection. Moreover, no additional prophylactic haemostatic manoeuvres are needed. Vasopressin, the main vasoconstricting agent proposed, has several reported side effects and its use avoided in many countries worldwide (Hobo et al., 2009).

## Conclusions

We report a simple and effective technique for laparoscopic cornual resection. Identifying the marginal zone and staying in it during dissection permits even the most junior laparoscopic surgeon to complete this operation with minimal blood loss concomitantly with minimal trauma to the adjacent myometrium.

## Video scan (read QR)

https://vimeo.com/910797672/d258363d71

**Figure:**